# Cera Flava Alleviates Atopic Dermatitis by Activating Skin Barrier Function via Immune Regulation

**DOI:** 10.3390/ijms22147531

**Published:** 2021-07-14

**Authors:** Gunhyuk Park, Byeong Cheol Moon, Goya Choi, Hye-Sun Lim

**Affiliations:** Herbal Medicine Resources Research Center, Korea Institute of Oriental Medicine, 111 Geonjae-ro, Naju-si 58245, Korea; gpark@kiom.re.kr (G.P.); bcmoon@kiom.re.kr (B.C.M.); serparas@kiom.re.kr (G.C.)

**Keywords:** atopic dermatitis, Cera Flava, house dust mite, immune regulation, skin barrier function

## Abstract

Cera Flava (CF), a natural extract obtained from beehives, is widely used in dermatological products owing to its wound healing, wrinkle reduction, UV-protective, and skin cell turnover stimulation effects. However, its effect on AD-like skin lesions is unknown. In this study, we used a mouse model of AD to evaluate the effects of CP at the molecular and phenotypic levels. Topical house dust mite (HDM) sensitization and challenge were performed on the dorsal skin of NC/Nga mice to induce AD-like cutaneous lesions, phenotypes, and immunologic responses. The topical application of CF for 6 weeks relieved HDM-induced AD-like phenotypes, as quantified by the dermatitis severity score, scratching frequency, and skin moisture. CP decreased immunoglobulin E, histamine, and thymic stromal lymphopoietin levels. Histopathological analysis showed that CF decreased epidermal thickening and the number of mast cells. CF attenuated HDM-induced changes in the expression of skin barrier-related proteins. Furthermore, CF decreased the mRNA levels of inflammatory factors, including interleukin (*IL*)*-1β, IL-4, IL-13, IL-8, TARC, MDC,* and *RANTES,* in dorsal skin tissue via the TLR2/MyD88/TRAF6/ERK pathway. CF influences skin barrier function and immune regulation to alleviate AD symptoms. It may therefore be an effective alternative to topical steroids for the treatment of AD.

## 1. Introduction

The skin is the largest organ of the human body and has multiple defensive and regulatory functions [1]. The outer layer of the epidermis, the stratum corneum, contributes to the barrier function of the skin. This epidermal barrier maintains cutaneous homeostasis and protects the body against numerous external stressors. Skin barrier dysfunction caused by endogenous or exogenous factors can lead to various disorders, such as atopic dermatitis (AD), xerosis cutis, and ichthyoses [2]. AD is a common cutaneous inflammatory disease resulting from the interaction between environmental and genetic factors related to epidermal barrier function [2,3]. The combination of barrier dysfunction and immune dysregulation contributes to the progression of AD. In particular, immune dysfunction and epidermal barrier distortion lead to immunoglobulin E (IgE) sensitization, a critical factor in skin inflammation [3]. Thymic stromal lymphopoietin (TSLP) is essential not only for skin allergic inflammation but also for allergen sensitization in impaired inflamed skin, and TSLP acts as a potent stimulator of type 2 helper T (Th2) cytokines, such as interleukin (IL)-4, 5, and 13, which in turn trigger IgE production [3]. Thus, AD therapies are usually directed toward restoring the skin barrier, limiting itching, and decreasing inflammation. Toll-like receptors (TLRs) are critical for immune and inflammatory responses in skin and are expressed in the various cells such as keratinocytes, Langerhans cells, macrophages resided in the skin [4]. TLRs play important roles in linking innate and adaptive immune responses to initiate immediate as well as long-term host defense mechanisms against danger signals. Dysregulation of TLRs is responsible for the pathogenesis of several inflammatory skin diseases [4,5]. Therefore, TLRs are a promising therapeutic target for skin diseases.

Cera Flava (CF), also known as “yellow beeswax” or “hwang-nap,” is a natural extract obtained by the heat compression filtration and purification of beehives. In traditional Korean medicine, CF is used in ointments to treat ulcers or wounds. The Dongui Bogam, the foremost classic of traditional Korean medicine, contains CF under the name “mil-nap” in the Chung-bu (category of medicinal materials derived from worms). CF is composed of a mixture of hydrocarbons, free fatty acids, monoesters, diesters, triesters, hydroxy monoesters, hydroxy polyesters, fatty acid polyesters, and unidentified compounds [6]. CF also contains small amounts of natural antibacterial agents and can prevent painful inflammation associated with infection. It is vitamin-rich, with abundant vitamin A, which improves wound healing, reduces wrinkles, protects the skin against UV radiation, and stimulates skin cell turnover [7,8]. However, scientific evidence for the use of CF in skin diseases, including AD, is still insufficient.

In this study, we evaluated the effect of CF on a mouse model of AD. In particular, NC/Nga mice were treated with the house dust mite (HDM), a common environmental allergen that causes AD in humans, to induce AD-like skin lesions, and the inhibitory effect of CF on the development of AD was evaluated.

## 2. Results

### 2.1. CF Improved AD Symptoms

**Figure 1:**
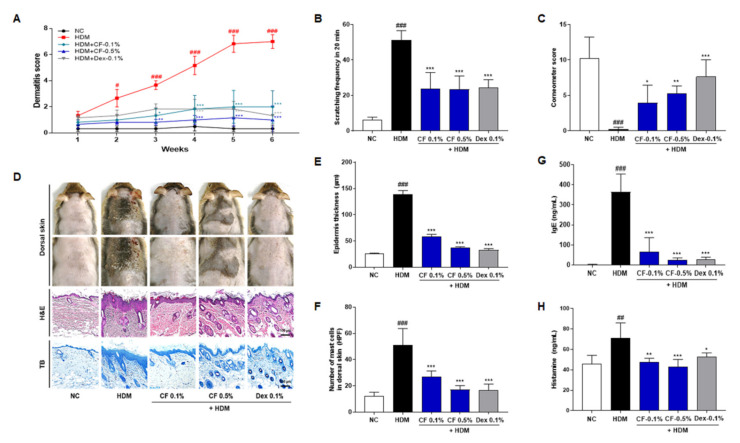
CF alleviates AD-like symptoms in the HDM-induced AD mouse model. (**A**) Dermatitis scores were evaluated weekly. (**B**) Scratching frequency and (**C**) skin moisture retention were evaluated on day 42 post-sensitization. (**D**) Histological features of mouse dorsal skin preparations. Tissues were excised, fixed in 10% formaldehyde, embedded in paraffin, and sectioned. Sections were stained with H&E (scale bar = 100 μm) or with TB to identify mast cells (scale bar = 100 μm). (**E**) Epidermal thicknesses in H&E-stained sections were measured under a microscope. (**F**) Mast cells were counted in toluidine blue-stained sections of dorsal skin tissues under a microscope. (**G**) IgE and (**H**) histamine serum levels were measured by ELISA. All data presented as means ± SEM for each group (n = 6 mice per group). Differences among groups were evaluated by one-way ANOVA, with Dunnett’s post-hoc tests; ^#^ *p* < 0.05, ^##^ *p* < 0.01 and ^###^ *p* < 0.001 compared with the NC group; * *p* < 0.05, ** *p* < 0.01, and *** *p* < 0.001 compared with the HDM group.

To assess the effect of CF on AD-like skin lesions, NC/Nga mice were topically treated with CF after the induction of AD-like skin lesions by HDM. The repeated topical application of HDM significantly increased the severity of AD and the scratching frequency and decreased skin moisture retention. Furthermore, the epidermis was thicker, and mast cell infiltration was greater in the HDM group than in the NC group. CF significantly attenuated HDM-induced changes in the scratching frequency, skin moisture retention, epidermal thickness, and mast cell infiltration, as well as attenuating the severity of AD (Figure 1A–F). Additionally, we evaluated the effect of CF on HDM-induced increases in IgE and histamine levels. CF attenuated the increased serum levels of IgE and histamine caused by HDM (Figure 1G,H).

### 2.2. CF Attenuated HDM-induced Changes in the Expression of Proteins Related to Skin Barrier Function

**Figure 2:**
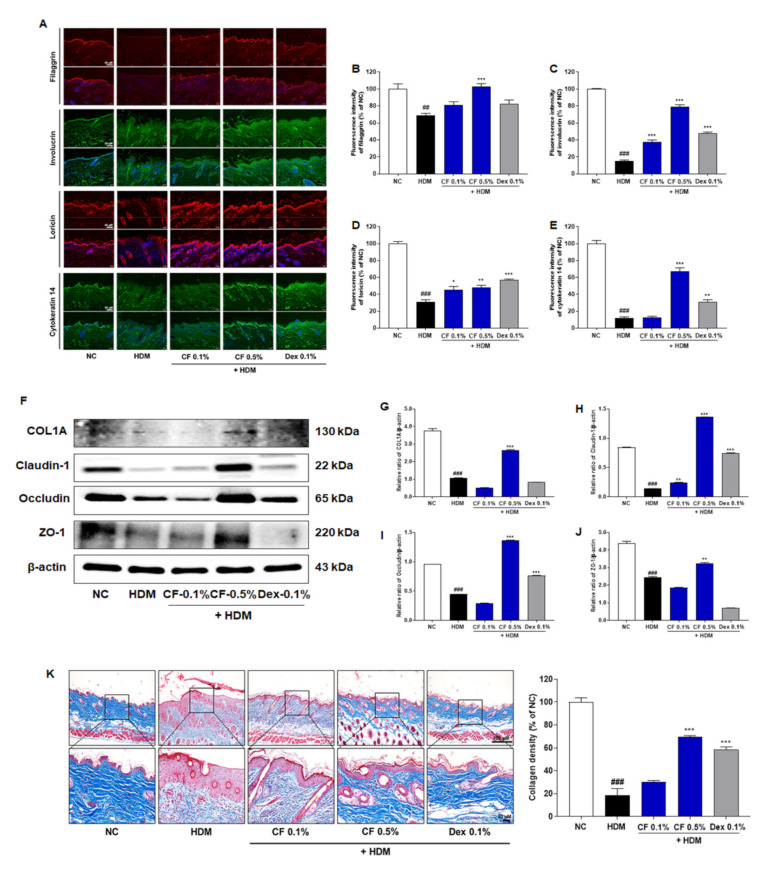
CF improves skin barrier function-related proteins and collagen formation in the HDM-induced AD mouse model. (**A**) Filaggrin, involucrin, loricrin, and cytokeratin 14 levels were evaluated by immunofluorescence in dorsal skin tissue. Scale bar = 50 μm. A quantitative analysis of dorsal skin tissue levels of (**B**) filaggrin, (**C**) involucrin, (**D**) loricrin, and (**E**) cytokeratin 14 levels. (**F**) COL1A, claudin-1, occludin, and ZO-1 were analyzed in dorsal skin tissue by western blotting. Bar graphs represent the relative expression levels of (**G**) COL1A, (**H**) claudin-1, (**I**) occludin, and (**J**) ZO-1. Relative expression levels were obtained by normalization against β-actin. (**K**) Representative micrographs of skin tissue sections stained with Masson’s trichrome for collagen after HDM induction for 6 weeks (Scale bar = 100 μm or 20 μm). Quantitative analysis of collagen density. All data are presented as means ± SEM (*n* = 6 mice per group). Statistical differences were evaluated by one-way ANOVA, with Dunnett’s post-hoc tests; ^##^ *p* < 0.01 and ^###^ *p* < 0.001 compared with the NC group; * *p* < 0.05, ** *p* < 0.01, and *** *p* < 0.001 compared with the HDM group.

To investigate the effect of CF on skin barrier function, we measured the expression levels of proteins associated with skin barrier function by immunofluorescence staining (Figure 2A). We found that the expression levels of filaggrin, involucrin, and loricrin in the dorsal skin tissue were higher in the CF-treated group than in the HDM-induced AD group (Figure 2B–D). Notably, the expression of cytokeratin 14 increased to a greater extent in mice treated with CF 0.5% than in those treated with CF 0.1% (Figure 2E). The protein expression of type I collagen (COL1A) increased significantly in mouse skin tissue after CF treatment (Figure 2F,G). Furthermore, the levels of proteins related to the skin barrier, including tight junctions, such as claudin-1, occludin, and zonula occludens-1 (ZO-1), were significantly higher in the CF 0.5% group than in the HDM-induced group (Figure 2F,H–J). MT staining was used to evaluate collagen formation in HDM-induced AD-like lesions. Compared with levels in the HDM group, collagen production was significantly higher in the CF groups (Figure 2K). 

### 2.3. CF Modulates the TLR2/MyD88/TRAF6/ERK Axis

**Figure 3:**
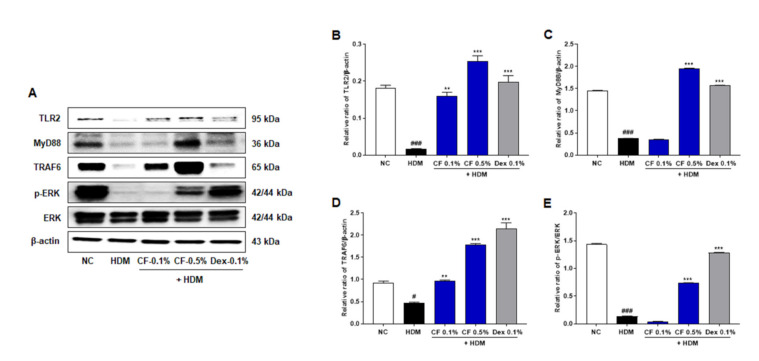
CF modulates TLR2/MyD88/TRAF6/ERK in the HDM-induced AD mouse model. (**A**) TLR2, MyD88, TRAF6, p-ERK were analyzed in dorsal skin tissue by western blotting. Bar graphs present the relative expression of (**B**) TLR2, (**C**) MyD88, and (**D**) TRAF6, and (**E**) p-ERK. Relative expression levels of TLR2, MyD88, and TRAF6 were obtained by normalization against β-actin. Relative expression levels of p-ERK were obtained by normalization against ERK. All data are represented per group, as means ± SEM (n = 6 mice per group). Differences were evaluated by one-way ANOVA, with Dunnett’s post-hoc tests; ^#^ *p* < 0.05 and ^###^ *p* < 0.001 compared with the NC group; ** *p* < 0.01 and *** *p* < 0.001 compared with the HDM group.

To investigate the regulatory mechanisms underlying the effects of CF in AD, we assessed the effect of CF on the expression of TLR2 and downstream molecules. The main adaptor molecules downstream of TLR2, such as MyD88 and TRAF6, are critical for activating downstream signaling pathways and inducing an inflammatory response. As shown in Figure 3, the protein levels of TLR2, MyD88, and TRAF6 decreased to a greater extent in the HDM group than in the NC group; however, levels were upregulated in the CF group in a dose-dependent manner. In addition, the expression level of phosphorylated ERK (p-ERK) was significantly higher in the CF group than in the HDM-induced AD group.

### 2.4. CF Inhibits the Expression of TSLP and Inflammatory Cytokines and Chemokines

**Figure 4:**
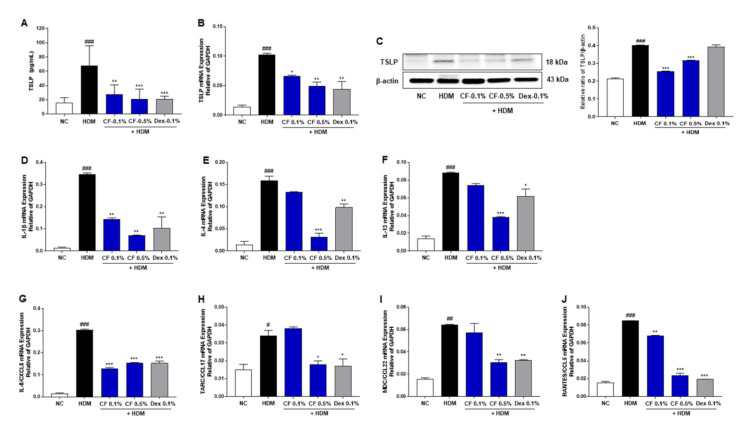
Effects of CF on the TSLP and inflammatory cytokines/chemokines in the HDM-induced AD mouse model. (**A**) TSLP serum levels were measured by ELISA. (**B**) The relative expression of TSLP was analyzed by real-time PCR in dorsal skin samples, with normalization against levels of *GAPDH*. (**C**) The expression of TSLP was detected by western blotting in dorsal skin samples. β-Actin was used as an internal control. The relative expression levels of inflammatory cytokines/chemokines were analyzed by real-time PCR in dorsal skin samples. Values were normalized against GAPDH expression levels. Normalized levels of the cytokines (**D**) IL-1β, (**E**) IL-4, and (**F**) IL-13 expression in dorsal skin samples are shown. Normalized levels of the chemokines (**G**) IL-8/CXCL8, (**H**) TARC/CCL17, (**I**) MDC/CCL22, and (**J**) RANTES/CCL5 expression in dorsal skin samples are shown. All data are presented as means ± SEM (n = 6 mice per group). Differences among groups were evaluated by one-way ANOVA, with Dunnett’s post-hoc tests; ^#^ *p* < 0.05, ^##^ *p* < 0.01, and ^###^ *p* < 0.001 compared with the NC group; ^*^ *p* < 0.05, ^**^ *p* < 0.01, and ^***^ *p* < 0.001 compared with the HDM group.

To investigate the effect of CF treatment on AD-related immune responses, we measured the production and expression of TSLP, an essential mediator of T cell maturation, in different groups of mice with AD. As shown in Figure 4A, the serum levels of TSLP in the HDM group were higher than those in the NC group; however, this upregulation was alleviated in the CF group in a dose-dependent manner. The mRNA and protein expression levels of *TSLP* were also significantly higher in the HDM group than in the NC group, while the CF group showed lower levels than the HDM group (Figure 4B,C). In addition, we examined the gene expression levels of *IL-1β, IL-4, IL-13, IL-8/C-X-C* motif chemokine ligand 8 (*CXCL8*), thymus and activation-regulated chemokine (*TARC*)/C-C motif chemokine ligand 17 (*CCL17*), macrophage-derived chemokine (*MDC*)/C-C motif chemokine ligand 22 (*CCL22*), and regulated activation, normal T cell expressed and secreted (*RANTES*)/C-C motif chemokine ligand 5 (*CCL5*). Noticeably, the mRNA levels of inflammatory cytokines and chemokines were higher in the dorsal tissue of mice in the HDM-induced AD-like symptom group than in the NC group. Topical CF treatment significantly reduced the mRNA levels of cytokines, such as *IL-1β, IL-4,* and *IL-13* (Figure 4D–F). In addition, the levels of inflammatory chemokines, including *IL-8/CXCL8, TARC/CCL17, MDC/CCL22,* and *RANTES/CCL5,* were downregulated in the CF group (Figure 4G–J).

## 3. Discussion

Since AD has a complex pathology, there are a variety of treatment options, including symptomatic therapy, the elimination of allergens, and the control of immune dysregulation. Topical steroids are the first-line treatment for AD; however, their abuse and misuse frequently cause side effects and steroid phobia [9]. Hence, natural extracts, which have fewer side effects than steroids, have gained significant interest for the treatment of AD. Natural extracts are important sources for therapeutic agents for AD-related diseases, although scientific evidence for their beneficial effects is insufficient [9,10]. We evaluated the effects of CF, a natural extract, on HDM-induced AD-like phenotypes in NC/Nga mice. Our results clearly demonstrated that the topical administration of CF could improve AD-like manifestations in an HDM-induced mouse model. CF influenced three major pathogenic factors in AD, namely pruritus, skin barrier abnormalities, and immunologic dysregulation.

Pruritus is an important clinical feature and a major diagnostic criterion for AD [11]. Controlling pruritus is important to interrupt the ‘itch–scratch cycle’ in AD, in which itchiness causes scratching, making the lesion itchier and more eczematous [11,12]. Scratching behavior aggravates the severity of AD, lowers quality of life, and causes psychological stress. In the present study, we found that CF effectively suppresses the development of HDM-induced AD and alleviates AD-like symptoms, such as scratching and a lack of moisture retention. As determined by H&E and TB staining of the dorsal skin, CF ameliorated hyperkeratosis and reduced the number of infiltrating mast cells in AD eczematous lesions, indicating that CF effectively improves AD. In addition, in pruritus, the activation of IgE by HDM resulted in the activation of mast cells, and cross-linking of IgE by HDM induced the release of histamine, one of the causes of itch in AD. We found that HDM-induced increases in serum concentrations of IgE were significantly attenuated by CF. Therefore, the repeated topical application of HDM exacerbated the symptoms of AD, such as skin lesions, inflammation, and mast cell infiltration, and these effects were ameliorated by CF treatment.

A defective skin barrier contributes to the progression of AD-like skin lesions by making it easy for allergens or irritants to penetrate the skin and induce an immunologic response [2,13]. Restoring a disrupted skin barrier is important for preventing AD development. Filaggrin is an important structural protein in the skin barrier that promotes the aggregation of the keratin cytoskeleton to build up the outermost epidermal barrier [14]. Involucrin and loricrin are natural moisturizing factors and are essential components of the epidermal envelope [14,15]. They are cross-linked to filaggrin and act as reinforcement proteins in the cornified envelope [15,16]. In the present study, the levels of filaggrin, involucrin, and loricrin were considerably reduced in the dorsal skin of mice in the HDM group, indicating that the epidermal barrier was impaired. CF treatment resulted in the recovery of epidermal protein expression to levels similar to those in the NC group. Cytokeratin 14 is an intermediate filament protein that is mainly expressed in the basal layer of healthy stratified epithelia [17]. In HDM-induced AD-like skin lesions, we observed the focal loss of cytokeratin 14 within the basal layer and in hair follicles; however, this loss was prevented by CF. Moreover, CF significantly promoted collagen production compared to the HDM-induced AD-like skin lesion. The increase in collagen density may be associated with tightening of the dermal extracellular matrix induced by the reduction of edema. Tight junctions reside below the stratum corneum and regulate selective permeability in the paracellular pathway [16]. Several studies have reported that filaggrin mutations result in reduced expression levels of tight junction proteins, including claudin-1, occludin, and ZO-1, and the disruption of tight junctions results in the incorporation of locus coeruleus dendrites into tight junctions and the processing of the immune response [15,18]. We observed that the expression levels of tight junction proteins, including cludin-1, occludin, and ZO-1, were lower in the HDM group than in the NC group. However, CF treatment increased the protein expression of claudin-1, occludin, and ZO-1 compared with levels in the HDM group, further indicating that CF restored skin barrier impairment and dysfunction. These results suggested that epithelial stabilization induced by CF may contribute to the restoration of skin barrier function in HDM-induced AD mice, and the suppression of HDM-induced inflammatory responses.

Immunologic dysregulation is a major factor in the pathogenesis of AD. TSLP can initiate cutaneous allergic responses during AD [19]. TSLP overexpression in the skin of transgenic mice results in AD-like manifestations, with dermal inflammatory cell infiltration and elevated serum IgE levels [19]. TSLP is highly expressed in the keratinocytes of patients with AD; it activates dendritic cells and induces Th2 responses [19,20]. Therefore, the suppression of TSLP may represent a novel therapeutic approach for AD. We found that CF treatment reduced TSLP levels. The effect of CF on TSLP levels supports the hypothesis that this natural extract has immunoregulatory properties. To further elucidate the mechanism underlying the anti-AD effects of CF, we investigated its effect on TLR2 and related proteins. Impaired TLR2 function is associated with the pathogenesis of AD [4,21]. Polymorphisms in TLR2 are associated with AD, and TLR2 was also found to be downregulated. In normal keratinocytes, TLR2 activation rapidly increases the expression of tight junction proteins in differentiated epidermal layers [21]. However, in AD, the expression of tight junction proteins is significantly reduced, indicating that TLR2 signaling is impaired in the suprabasal layers of the epidermis, where these genes are expressed [22]. These receptors are involved in ERK activation and mediate multiple keratinocyte responses [22,23]. We observed lower levels of TLR2, MyD88, TRAF6, and phosphorylated ERK in the HDM group than in the NC group. However, CF treatment increased the protein expression levels of TLR2, MyD88, TRAF6, and phosphorylated ERK compared with levels in the HDM group. Impaired TLR2 function promotes the loss of barrier integrity and immune system imbalance during the acute phase of AD. However, the aberrant activation of TLR2 may lead to T helper cell-related immune responses during the chronic phase of AD and the production of keratinocyte-specific cytokine TSLP, which drives allergic immune responses. Therefore, strategies that finely modulate TLR2 expression or function hold promise for the restoration of barrier function and the immune balance in AD.

Cytokines produced by Th2 lymphocytes, including IL-4 and IL-13, are central to the pathogenesis of AD [11]. TSLP is a highly expressed cytokine in epidermal keratinocytes in AD and has been recognized as the master regulator linking the innate response at the barrier surface to the Th2-skewed adaptive immune response in AD [19]. Other chemokine variants have also been identified in AD, including IL-8, TARC, and MDC. RANTES polymorphisms are associated with allergen sensitization [24,25]. In this study, CF treatment normalized the Th2-mediated immunologic dysregulation in HDM-induced AD-like phenotypes in NC/Nga mice. CF ameliorated the effect of HDM by significantly reducing the mRNA levels of *IL-1β, IL-4, IL-13, IL-8, TARC, MDC*, and *RANTES* in the mouse dorsal skin.

Beeswax is naturally yellow in colour (CF), but becomes white when purified or bleached [26]. CF can cause contact allergy, and some cases of contact cheilitis, as well as cosmetic and occupational contact dermatitis caused by CF, have been described [27]. It is not known whether there is any difference in allergenicity between white and yellow beeswax [27,28]. Moreover, the causative haptens in beeswax remain to be identified, and the relationship between contact allergy to propolis and contact allergy to beeswax remains to be characterized [28,29].

## 4. Material and Methods

### 4.1. Preparation of the CF Extract

CF was purchased from Ukherb (Busan, Korea) and authenticated by Dr. Goya Choi (Herbal Medicine Resources Research Center, Korea Institute of Oriental Medicine, Naju, Korea). A CF voucher specimen (#2-18-190) was deposited at the Herbal Medicine Resources Research Center, Korea Institute of Oriental Medicine. CF was extracted in distilled water for 3 h under reflux at 100 ± 2 °C. The extract was filtered, evaporated on a rotary vacuum evaporator, and freeze-dried. The powder was then stored at 4 °C.

### 4.2. Experimental Animals

Male NC/Nga mice (8 weeks old) were purchased from Central Laboratory Animal Inc. (Seoul, Korea) and maintained under temperature- and light-controlled conditions (22 ± 2 °C and 12 h light–dark cycle), with food and water provided ad libitum. All experimental procedures were approved by the Korea Institute of Oriental Medicine Animal Care and Use Committee (approval number: #19-039) and were performed in accordance with the relevant guidelines and regulations.

### 4.3. Induction of Experimental AD

To induce barrier integrity disruption, 200 μL of 4% SDS solution was applied to the shaved dorsal skin and ventral and dorsal ear surfaces of NC/Nga mice. Experimental AD-like skin lesions were induced by the topical application of 50 mg of HDM ointment containing Dermatophagoides farinae extract (Biostir-AD; Biostir Inc., Osaka, Japan) twice per week for 6 weeks. The mice were divided into five groups (n = 6 per group): (1) negative control (NC) group (vehicle-treated mice, distilled water), (2) HDM group (HDM + distilled water), (3) CF 0.1% group (HDM + CF 0.1%), (4) CF 0.5% group (HDM + CF 0.5%), and (5) Dex 0.1% group (HDM + dexamethasone [Dex] 0.1%). CF and Dex (Sigma–Aldrich, St. Louis, MO, USA) were dissolved in distilled water and topically applied every day during HDM sensitization for 6 weeks. Dex was used as the positive control.

### 4.4. Evaluation of Dermatitis Scores

The relative severity of dermatitis was assessed macroscopically, according to the Eczema Area and Severity Index scoring system as follows: 0, no symptoms; 1, mild symptoms; 2, moderate symptoms; 3, severe symptoms. The dermatitis scores were defined as the sum of scores for erythema/hemorrhage, edema, excoriation/erosion, and scaling/dryness [30]. Mice were photographed once each week for 6 weeks using a digital camera (Canon EOS 5D Mark IV; Canon Inc., Tokyo, Japan), and clinical evaluations were based on these images.

### 4.5. Evaluation of Scratching Behavior

Scratching behavior was evaluated in the context of HDM sensitization tests, performed as previously reported [31], and AD-indicative behavioral changes, such as scratching for periods of longer than 20 min, were measured and recorded.

### 4.6. Histological Analysis and Measurement of Skin Thickness and Infiltrating Mast Cells

To evaluate tissue architecture, sections were stained with hematoxylin and eosin (H&E) solution (Sigma-Aldrich) and mounted under cover slips using Dako mounting medium (Dako Cytomation, Glostrup, Denmark). Epidermal thickness was quantified by microscopic examination. Briefly, 10 randomly selected areas were observed in H&E-stained sections (three sections per animal) under a microscope (Olympus Microscope System CKX53; Tokyo, Japan). To measure the degree of mast cell infiltration, sections were stained with toluidine blue (TB), and mast cells were counted in four fields of view under a microscope (Olympus Microscope System CKX53). Collagen density was measured using the Masson’s Trichrome (MT) Stain Kit (Vitro Vivo Biotech, Rockville, MD, USA), according to the manufacturer’s protocol. All measurements and quantitative analyses were performed according to a previously published method [31].

### 4.7. Evaluation of Moisture Retention

Moisture retention in the dorsal skin of mice was measured 42 days after the initiation of treatment, at 22 ± 2 °C and 50–55% humidity, using a skin evaporative water recorder (Tewameter TM300; Courage-Khazaka Electronic, Koln, Germany) and a corneometer (Courage-Khazaka Electronic). Values were recorded only after signal stabilization, which occurred approximately 10 s after the probe was placed on the skin.

### 4.8. Measurement of Serum IgE, Histamine, and TSLP Levels

Serum levels of IgE (BioLegend, San Diego, CA, USA), histamine (Oxford Biomedical Research, Rochester Hills, MI, USA), and TSLP (R&D Systems Inc., Minneapolis, MN, USA) were measured using commercial ELISA kits, according to the manufacturers’ instructions.

### 4.9. Quantification of Filaggrin, Involucrin, Loricrin, and Cytokeratin 14 by Immunofluorescence

Dorsal skin sections were rinsed briefly in PBS and treated with 0.5% BSA for 30 min. The dorsal skin tissue on the slide was incubated with rabbit anti-filaggrin (1:500 dilution; GeneTex Inc., Irvine, CA, USA), mouse anti-involucrin (1:500 dilution; Invitrogen, Carlsbad, CA, USA), rabbit anti-loricrin (1:500 dilution; Abcam, Cambridge, UK), and mouse anti-cytokeratin 14 (1:500 dilution; Abcam) overnight at 4 °C in the presence of 0.3% Triton X-100 and normal goat serum. The slides were then incubated for 2 h with an Alexa Fluor-conjugated secondary antibody (diluted 1:500). The dorsal skin tissue on the slide was washed in PBS and mounted using Vectashield mounting medium, containing 4′,6-diamidino-2-phenylindole. Images were obtained using a fluorescence microscope (Olympus Microscope System CKX53). A threshold for positive staining was determined for each image that included processes but excluded background staining. Dorsal skin tissue regions were analyzed quantitatively using ImageJ 1.50i (National Institutes of Health).

### 4.10. Western Blot Analysis

Dorsal skin tissue was lysed in RIPA lysis and extraction buffer (Thermo Scientific, Rockford, IL, USA), containing a protease inhibitor cocktail (cOmplete™; Roche, Mannheim, Germany). Anti-aquaporin 3, anti-TSLP (all from Abcam), anti-filaggrin, anti-cytokeratin 14, anti-p-extracellular-signal-regulated kinase (ERK), anti-ERK, anti-p-AKT, anti-AKT (all from Cell Signaling Technology, Danvers, MA, USA), anti-TLR2, anti-MyD88, anti-TRAF6 (all from Invitrogen), and anti-β-actin (Santa Cruz Biotechnology, Dallas, TX, USA) antibodies were used. Western blot analysis was performed according to a previously published method [31].

### 4.11. RNA Extraction and Real-Time Reverse Transcription PCR

Dorsal skin tissue was homogenized using TRIzol reagent (Invitrogen). RNA extraction and real-time PCR were performed according to a previously published method [31]. Primer sequences were as follows: *TARC/CCL17* (forward 5′-attcaaaaccagggtgtctcc-3′, reverse 5′-ctcttgttgttggggtccga-3′), *MDC/CCL22* (forward 5′-gcgtggtgttgctaaccttc-3′, reverse 5′-ggccacggtcatcagagtag-3′), and *RANTES/CCL5* (forward 5′-ccccatattcctcggacacc-3′, reverse 5′-gacaaagacgactgctgggt-3′).

### 4.12. Statistical Analysis

Data are presented as group means ± SEM. All of the experiments were performed at least 3 times. Statistical analysis was performed using GraphPad Prism 7.0 (GraphPad Software, San Diego, CA, USA). Comparisons were performed using one-way analysis of variance with Dunnett’s post-hoc tests. Differences were considered statistically significant at *p* < 0.05.

## 5. Conclusions

**Figure 5:**
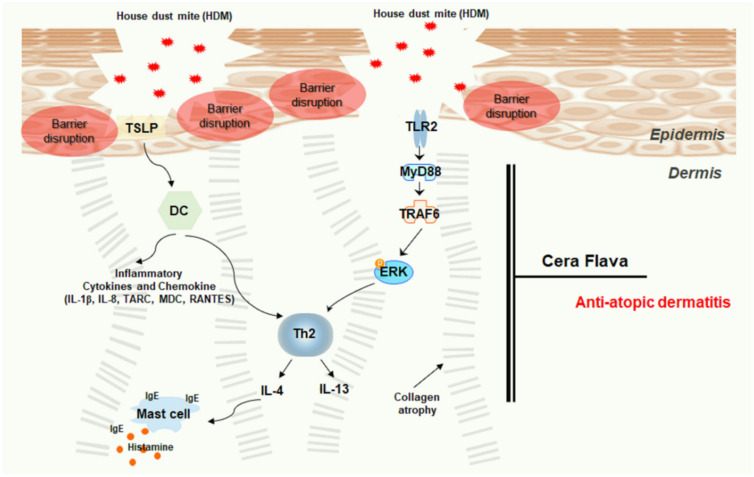
Schematic model of the effects of CF treatment on the HDM-induced AD mouse model. Upon HDM exposure, epithelial barrier disturbances, functional defects, and inflammatory cell infiltration were observed. The barrier-disrupted epidermis released abundant TSLP, which promotes cytokine/chemokine immune responses. CF treatment reduced AD severity, relieved AD-like symptoms, and attenuated inflammatory cell infiltration and cytokine/chemokine expression by controlling the TLR2/MyD88/TRAF6/ERK axis. CF alleviates AD by activating skin barrier function via immune regulation.

HDM-mediated TLR2/ERK downregulation in AD not only suppressed the production of inflammatory cytokines/chemokines but also enhanced the tight junction barrier function of the epidermis. We demonstrated that CF efficiently attenuated HDM-induced AD-like phenotypes in NC/Nga mice, relieving pruritus, restoring skin barrier, and normalizing immune function (Figure 5). Thus, CF is a promising candidate for the treatment of AD. However, TSLP can be induced by the activation of TLR2 or by TLR-independent mechanisms, and it is still unclear whether the aberrant activation of TLR2 contributes to high TSLP expression in AD. Further studies are needed to define the role of TLR2 in TSLP expression and to convert AD from a Th2-dominant acute phase to a Th2-Th1 mixed chronic inflammation phase. In addition to this study of anti-atopic dermatitis of CF, clinical studies on potential allergic properties in future CF, and interrelationships with anti-atopic dermatitis should also be carried out.

## Data Availability

Data sets are available by request to the corresponding author.

## Abbreviations:

AD	atopic dermatitis
CCL	C-C motif chemokine ligand
CF	cera flava
CXCL8	C-X-C motif chemokine ligand 8
Dex	dexamethasone
ERK	extracellular-signal-regulated kinase
HDM	house dust mite
H&E	hematoxylin and eosin
IgE	immunoglobulin E
IL	interleukin
MDC	macrophage-derived chemokine
MT	Masson’s trichrome
RANTES	regulated activation, normal T cell expressed and secreted
TARC	thymus and activation-regulated chemokine
TB	toluidine blue
TBST	Tris-buffered saline containing Tween 20
Th1	type II helper T cell
Th2	type II helper T cell
TLR	toll-like receptor
TSLP	thymic stromal lymphopoietin
ZO-1	zonula occludens-1
